# Enhancements to the ADMIXTURE algorithm for individual ancestry estimation

**DOI:** 10.1186/1471-2105-12-246

**Published:** 2011-06-18

**Authors:** David H Alexander, Kenneth Lange

**Affiliations:** 1 Department of Biomathematics, UCLA, Los Angeles, California, USA; 2 Department of Human Genetics, UCLA Los Angeles, California, USA; 3 Department of Statistics, UCLA Los Angeles, California, USA

## Abstract

**Background:**

The estimation of individual ancestry from genetic data has become essential to applied population genetics and genetic epidemiology. Software programs for calculating ancestry estimates have become essential tools in the geneticist's analytic arsenal.

**Results:**

Here we describe four enhancements to ADMIXTURE, a high-performance tool for estimating individual ancestries and population allele frequencies from SNP (single nucleotide polymorphism) data. First, ADMIXTURE can be used to estimate the number of underlying populations through cross-validation. Second, individuals of known ancestry can be exploited in supervised learning to yield more precise ancestry estimates. Third, by penalizing small admixture coefficients for each individual, one can encourage model parsimony, often yielding more interpretable results for small datasets or datasets with large numbers of ancestral populations. Finally, by exploiting multiple processors, large datasets can be analyzed even more rapidly.

**Conclusions:**

The enhancements we have described make ADMIXTURE a more accurate, efficient, and versatile tool for ancestry estimation.

## 1 Background

Our program ADMIXTURE estimates individual ancestries by efficiently computing maximum likelihood estimates in a parametric model. The model [1,2] posits that genotype *n_ij _*for individual *i *at SNP *j *represents the number of type "1" alleles observed. Given *K *ancestral populations, the success probability  in the binomial distribution *n_ij _*~ Bin(2, *p_ij _*) depends on the fraction *q_ik _*of *i*'s ancestry attributable to population *k *and on the frequency *f_kj _*of allele 1 in population *k*. ADMIXTURE maximizes the biconcave log-likelihood(1)

of the model using block relaxation. The alternating updates of the parameter matrices *Q *= (*q_ik_*) and *F *= (*f_kj_*) both rely on sequential quadratic programming. Convergence is accelerated by applying a quasi-Newton extrapolation algorithm [3]. Further details of our core algorithm are documented elsewhere [4]. The performance of ADMIXTURE is compelling. An ADMIXTURE analysis is typically three to four orders of magnitude faster than a comparable STRUCTURE [1] analysis.

The advanced features of ADMIXTURE described here allow the user to automate the choice of the number of underlying populations *K *and to exploit known ancestral populations in a supervised learning mode. Our penalized estimation mechanism can provide many of the benefits of a Bayesian analysis at a fraction of the computation time. These features make ADMIXTURE a suitable replacement for STRUCTURE in most practical applications. Given the ever-increasing size of genotype datasets, the inherent speed of our optimization algorithm, coupled with the parallel-processing mode described here, may render ADMIXTURE the *only *viable model-based ancestry analysis tool for many users.

## 2 Implementation

### Cross-validation

The choice of the number of ancestral populations *K *can prove difficult when the underlying population genetics of a species is poorly understood. STRUCTURE provides a means of estimating the best value of *K *by computing the *model evidence *for each *K *from a range of choices. The model evidence is defined as(2)

where *f *represents the data likelihood and *π *represents a prior density on the parameters. STRUCTURE approximates the integral via Monte Carlo methods. Our optimization framework is not well suited to evaluating this integral. As an alternative, we employ cross-validation. In cross-validation, we aim to identify the best *K *value as judged by prediction of systematically withheld data points. A similar tactic is also employed by the haplotype analysis program fastPHASE [5] and is inspired by Wold's method for cross-validating PCA models [6].

Our *v*-fold cross-validation procedure partitions the non-missing genotypes into *v *roughly equally sized subsets (*folds*). At each of *v *iterations, the members of one of the folds are masked (temporarily marked as missing) to yield a new data matrix  Analysis of the masked data matrix  poses no new challenges. In computing the log-likelihood, score, and observed information matrix of , we simply ignore the entries (*i*, *j*) with missing values. Maximization of the log-likelihood readily yields new estimates  and  for the masked data. We then predict each masked value *n_ij _*by . Prediction error is estimated by averaging the squares of the deviance residuals for the binomial model [7],(3)

across all masked entries over all folds. Minimizing this estimated prediction error on a grid of *K *values then suggests the most suitable *K*.

### Supervised learning of admixture coefficients

ADMIXTURE's strategy of simultaneously estimating individual ancestry fractions *Q *and population allele frequencies *F *is ideal when nothing is known about the contributing ancestral populations. In many scenarios, however, these populations are known and several reference individuals from each population are available. Here it is of interest to estimate the potentially admixed ancestries of the remaining individuals. We term this *supervised *analysis, as the reference individuals furnish training samples in a supervised learning context. To perform supervised analysis in ADMIXTURE, an .ind file mapping individuals to populations must be provided, and the flag --supervised must be attached to the command line.

Ancestry estimates can be estimated more accurately in supervised analysis because there is less uncertainty in allele frequencies. Interpretation of results is simplified, and run times are shorter owing to the reduced number of parameters to estimate. Both the number of iterations until convergence and the computational complexity per iteration decrease. However, we caution that supervised analysis is only suitable when the reference individuals can be assigned to ancestral populations with certainty and ancestral populations are fairly homogeneous. For exploratory analyses, unsupervised analysis is more appropriate and therefore remains the default in ADMIXTURE.

### Penalized estimation and model parsimony

As noted in our later comparison of supervised and unsupervised learning, datasets culled from closely related populations typed at a modest numbers of SNPs can pose substantial challenges in ancestry estimation. For instance, overfitting tends to yield ancestry estimates with inflated amounts of admixture. The Bayesian solution to this problem is to impose an informative prior to steer parameter estimates away from danger when data is sparse. Thus, STRUCTURE imposes Dirichlet prior distributions on ancestry parameters and estimates a hyperparameter *α *that controls the strength of the prior distributions.

A suitable alternative in our optimization framework is to perform penalized estimation. Rather than maximizing the log-likelihood, we maximize an objective function  consisting of the log-likelihood minus a penalty . The penalty is designed to discourage the undesirable biases in the estimated ancestry matrix  just mentioned. The tuning constant *λ *controls the strength of the penalty. While it is tempting to consider the negated logarithm of the Dirichlet prior density appearing in STRUCTURE as a penalty, the Dirichlet(*α*, ..., *α*) density is unbounded above in the parameter regime *α *< 1--arguably the most useful setting for the *α *parameter--and is therefore unusable in our optimization framework. A better alternative is the approximate *ℓ*_0 _penalty [8]

which encourages not only shrinkage but also aggressive parsimony. In particular, the approximate *ℓ*_0 _penalty drives small admixture coefficients to zero. Parsimony is desirable because it leads to more easily interpretable and probably more realistic parameter estimates. Estimation is performed by maximizing  over its arguments. Increasing *λ *or the second tuning constant *γ *elevates the extent of shrinkage and parsimony in the resulting estimates  and .

Determination of the penalty tuning constants *λ *and *γ *is nontrivial. In our hands cross-validation has proved effective on simple simulated datasets. The tuning constants *λ *and *γ *are user-defined options, so users can explore different settings consistent with cross-validation or their own heuristics.

### Exploiting Multiple Processors

Very large datasets (millions of SNPs, thousands of individuals) can reduce even ADMIXTURE's efficient algorithms to a crawl. Since our original publication, we have tuned our core algorithm and improved its speed by a factor of two. We have also implemented a parallel execution mode that lets ADMIXTURE exploit multiple processors. This new option employs the OpenMP [9] framework designed for simple parallelization using compiler #pragma directives. To perform analyses with, for example, four threads, the user need only add the flag -j4 to the command line. Hence

$ admixture Data/hapmap3.bed 3 -j4

analyzes the data file hapmap3.bed using 4 threads, assuming *K *= 3 ancestral populations. Analyses of our hapmap3 dataset with *K *= 3 were accelerated by 392% on a four processor machine.

## Results and Discussion

### The effectiveness of cross-validation

**Figure 1:**
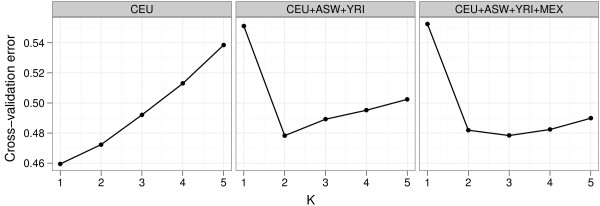
**Cross-validation (CV) of three datasets derived from the HapMap 3 resource using *v *= 5 folds**. After subsampling 13,928 markers to minimize linkage disequilibrium, we separately cross-validated datasets containing unrelated individuals from the (a) CEU, (b) CEU, ASW, and YRI, and (c) CEU, ASW, YRI, and MEX HapMap 3 subsamples. Plots display CV error versus *K*. CV for the CEU dataset suggests *K *= 1 is the best fit, agreeing with intuition; *K *= 2 is the best fit for the CEU+ASW+YRI dataset, which contains European, African, and admixed African-American samples; *K *= 3 is the best fit for CEU+ASW+YRI+MEX, which additionally contains Mexican-Americans.

Figure 1 demonstrates the effectiveness of cross-validation on several datasets culled from HapMap 3 [10]. For these datasets, cross-validation was able to accurately identify the number of ancestral populations. While we have not performed extensive simulation studies, our experience has shown that the success of cross-validation depends in part on the degree of differentiation between the populations under study as quantified by Wright's fixation index *F_ST _*. Very closely related populations cannot be accurately separated. We speculate that this phenomenon may have a theoretical connection to the "phase-change" phenomenon observed by Patterson et al. [11]. For a dataset of fixed dimensions, they note that the *F_ST _*value separating two populations must exceed a certain threshold before the population samples can be reliably distinguished in principal component analysis.

### Supervised analysis can yield more precise estimates

To explore the benefits of supervised analysis, we generated a number of artificial datasets and evaluated the empirical precision of parameter estimates compared to the true *Q *and *F*. The ancestral allele frequencies *F *were first generated using the Balding-Nichols model [12] for 10,000 markers in each of two populations differentiated by an *F_ST _*value of .01 (comparable to the genetic distances observed between closely related populations within a continent) and with ancestral allele frequencies drawn uniformly from [0, 1]. Then, for each of 100 datasets, 400 individuals were simulated using ancestries fixed as follows: one hundred individuals with ancestry entirely from population 1, one hundred individuals from population 2, and the remaining two hundred with admixed ancestries spaced uniformly on a grid between population 1 and population 2. Supervised and unsupervised ADMIXTURE analyses performed on these datasets revealed several interesting patterns. First, supervised analysis more accurately recovered the underlying allele frequencies. On average the root-mean-squared error in estimating the vector *f*_1 _of reference allele frequencies for population 1 was .046 for unsupervised analysis but .040 for supervised. In general, it appears that errors in estimating *F *cause overestimation of the *F_ST _*between the ancestral populations. Indeed, here the average *F_ST _*estimate of .024 for unsupervised analysis fell to .019 for supervised analysis (true *F_ST _*of .010).

**Figure 2:**
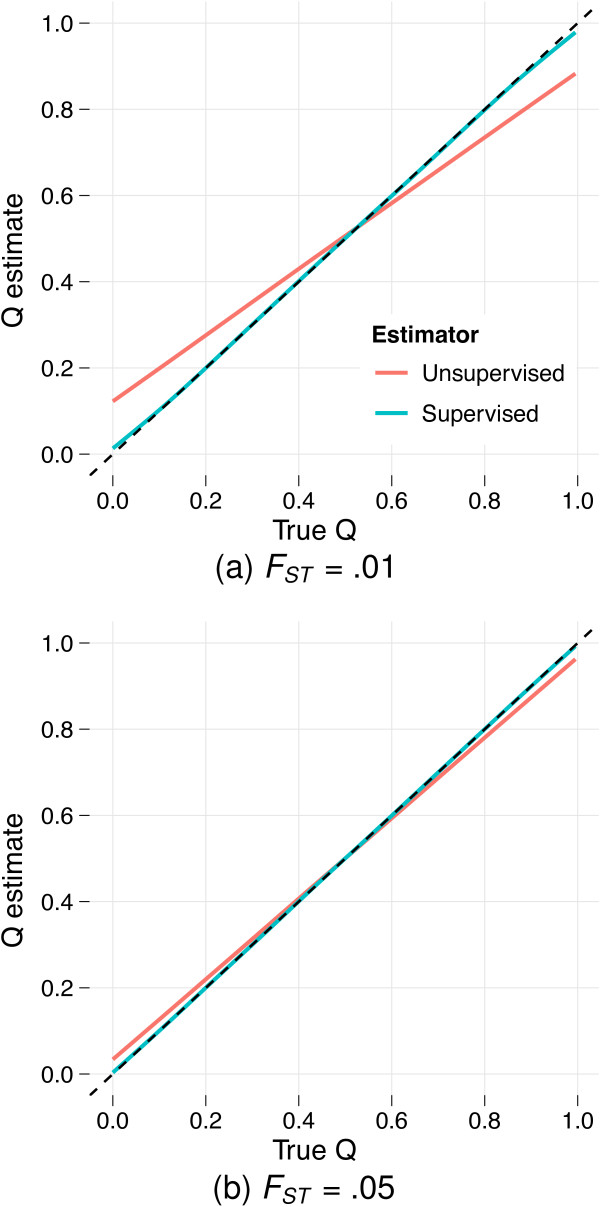
**Errors in estimating ancestral allele frequencies lead to bias in estimating ancestry fractions (*Q*), with many individuals ascribed too much admixture**. The plot shows an estimate of the relationship  between the true ancestry fraction *q_i_*_1 _(fraction of ancestry attributed to population 1) and the resulting estimate  as determined via a nonparametric regression (LOESS) model fitted to the results from analyses of 100 simulated datasets. Reference individuals are excluded from the plots and regression analyses. The dotted line *y *= *x *is tracked closely by the conditional mean of supervised estimates, suggesting little bias. However, in panel (a) (simulations with *F_ST _*= .01) the conditional mean of the unsupervised estimates deviates substantially, exhibiting an upward bias for low *q_i_*_1 _and a downward bias for high *q_i_*_1_. The bias is mitigated using simulations with *F_ST _*= .05, as shown in panel (b), or by using a larger number of markers (*J *= 300, 000, not shown).

The flip-side of the systematic overestimation of the separation between populations is that ancestry fraction estimates suffer from bias. In particular, individuals will be ascribed a greater degree of admixture than they actually possess. Figure 2 illustrates this effect. Individuals with low *q_i_*_1_, reflecting a small degree of ancestry from population 1, have upward-biased estimates , while estimates for those with high *q_i_*_1 _exhibit a downward bias. The net effect is an apparent bias towards ancestry fractions of .5. Supervised analysis appears not to suffer from this bias.

In our opinion the apparent bias in unsupervised ancestry estimates should not be cause for alarm. The bias becomes much less prominent for larger datasets or datasets where the ancestral populations are better differentiated. Performing the same simulation with an *F_ST _*of .05, the bias in *Q *estimates is mitigated substantially, as seen in Figure 2b. A similar effect is apparent when we increase the number of markers *J *to 100,000 or more.

Hence, it is evident that supervised analysis, when applicable, can yield more precise estimates that are less susceptible to the biases seen in unsupervised analysis. Another benefit of supervised analysis is that it runs considerably faster. For the 10 simulated datasets with 10,000 markers, supervised analysis took an average of 5.15 seconds, while unsupervised analysis averaged 27.5 seconds.

### The effects of penalized estimation

**Figure 3:**
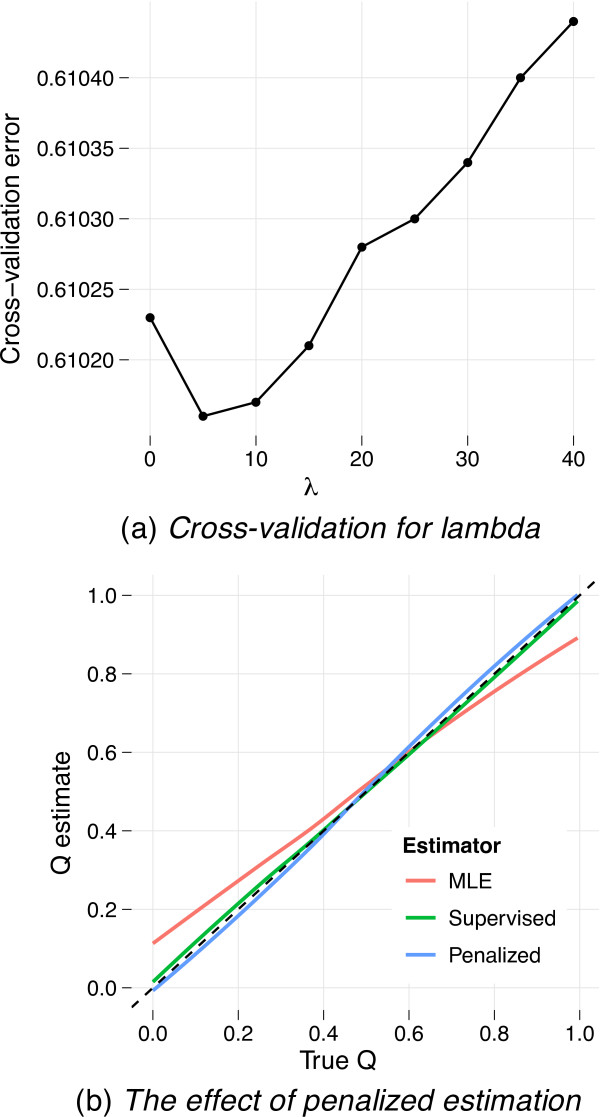
**Penalized estimation can reduce the bias in ancestry estimates that appears for small marker sets or closely related ancestral populations**. We applied penalized estimation to the simulated dataset of 10,000 SNP markers from admixed individuals from two populations differentiated by *F_ST _*= .01. Panel (a) shows that 5-fold cross-validation selects *λ *= 5 as the optimal strength of penalization. The results of penalization with *λ *= 5 are compared, in panel (b), with the maximum likelihood (unsupervised) estimates and with the supervised estimates, all visualized via nonparametric regression as in Figure 2. Reference individuals are excluded from the regression models.

The bias in ancestry estimates observed in Figure 2 is principally a problem for small datasets with closely related ancestral populations. Nevertheless, we designed our penalized estimation procedure partly to reduce this bias. To demonstrate the effectiveness of penalization, we explored penalized estimation in the context of the previous simulation of admixed individuals from two populations differentiated by *F_ST _*= .01. Fixing *γ *= .1 and performing cross-validation on a single one of these simulated datasets for *λ *values spaced between 0 and 100, we identified *λ *= 5 as the value minimizing cross-validation error (Figure 3a). Comparing the ancestry estimates with those from maximum likelihood unsupervised and supervised analyses (Figure 3b) reveals that penalized estimation mitigates bias substantially.

## Conclusion

ADMIXTURE is a fully-featured, highly efficient, and easy-to-use tool for ancestry estimation from SNP datasets. The four enhancements described here promote great flexibility in both exploratory and focused studies of genetic ancestry. Cross-validation enables rational choice of the number of ancestral populations. Supervised analysis mode can yield more accurate ancestry estimates when the number and makeup of contributing populations are certain. Parallelizing the code reduces run times and allows more ambitious analyses involving more people and SNPs. Finally, penalizing weak evidence for admixture promotes model parsimony and yields ancestry fractions more in line with users' expectations.

## Availability and requirements

**Project name: **ADMIXTURE

**Project home page: **http://www.genetics.ucla.edu/software/admixture; snapshot of software available as Additional File 1.

**Operating systems: **Linux, Mac OS X

**Programming languages: **C++

**Other requirements: **None

**License: **Binaries freely available; source code proprietary

**Any restrictions to use by non-academics: **None

## Competing interests

The authors declare that they have no competing interests.

## Authors' contributions

DHA and KL devised the algorithms for penalized estimation, cross-validation, supervised analysis, and parallel execution. DHA implemented the software. DHA and KL designed the experiments, which DHA then executed and analyzed. DHA and KL composed the manuscript. The authors have approved the final manuscript.

## Supplementary Material

Additional file 1 **Software.zip, a zip archive containing Mac OS X and Linux executables, is a snapshot of the ADMIXTURE software at the time of submission of this manuscript**. The current version is maintained at http:///www.genetics.ucla.edu/software/admixture. Click here for file
