# Supplementary material for: Enhancements to the ADMIXTURE algorithm for individual ancestry estimation
Source: BMC Bioinformatics. 2011 Jun 18;12:246. doi: 10.1186/1471-2105-12-246 (PMC3146885; doi:10.1186/1471-2105-12-246)
Supplement: Additional file 1 — Software.zip, a zip archive containing Mac OS X and Linux executables, is a snapshot of the ADMIXTURE software at the time of submission of this manuscript. The current version is maintained at http:///www.genetics.ucla.edu/software/admixture. [file 1471-2105-12-246-S1.ZIP › Software/admixture-manual.pdf]

ADMIXTURE 1.1 Software Manual

David H. Alexander John Novembre Kenneth Lange

February 8, 2011

Contents

1 Quick start **1**

2 Reference **3**
2.1 How do I choose the correct value for K? 3
2.1.1 Background 4
2.2 How do I plot the Q estimates? 5
2.3 Do I need to thin the marker set for linkage disequilibrium? 5
2.4 How many markers do I need to supply to ADMIXTURE? 6
2.5 How do I change the random seed?' 6
2.6 Optimization Methods 7
2.7 Termination criteria 7
2.8 Acceleration methods 8
2.9 Bootstrapping 8
2.10 Supervised analysis 8
2.11 Multithreaded mode 9
2.12 Penalized estimation 9

3 Citation and further information **9**

1 Quick start

ADMIXTURE is a program for estimating ancestry in a model-based manner from large autosomal SNP genotype datasets, where the individuals are unrelated (for example, the individuals in a case-control association study).

ADMIXTURE's input is binary PLINK (`.bed`), ordinary PLINK (`.ped`), or EIGENSTRAT (`.geno`) formatted files and its output is simple space-delimited files containing the parameter estimates.

To use ADMIXTURE, you need an input file and an idea of K, your belief of the number of ancestral populations. You should also have the associated support files alongside your main input file, in the same directory. For example, if your primary input file is a `.bed` file, you should have the associated `.bim` (binary marker information file) and `.fam` (pedigree stub file) files in the same directory. If your primary input file is a `.ped` or `.geno` file, a corresponding PLINK style `.map` file should be in the same directory.

If you have an binary PED (`.bed`) formatted file in your current directory

```
% ls
hapmap3.bed hapmap3.bim hapmap3.fam
```

and you believe that the individuals in the sample derive their ancestry from three ancestral populations then run admixture like this:

```
% admixture hapmap3.bed 3
```

ADMIXTURE will start running. Hopefully it will finish soon, and it will then output some estimates:

```
% ls
hapmap3.bed hapmap3.bim hapmap3.fam hapmap3.3.Q hapmap3.3.F
```

There is an output file for each parameter set: Q (the ancestry fractions), and F (the allele frequencies of the inferred ancestral populations). Note that the output filenames have '3' in them. This indicates the number of populations (K) that was assumed for the analysis. This filename convention makes it easy to run analyses using different values of K in the same directory.

If you have a PLINK .ped "12" coded file—generated by a command like

```
% plink --file hapmap --recode12 --out hapmap
```

—then you follow basically the same instructions:

```
% admixture hapmap3.ped 3
```

Note that ADMIXTURE infers the format of your input file based on the file extension (.bed, .ped, or .geno).

You can run ADMIXTURE on a input file located in another directory, but its output will always be put in the current working directory. For example:

```
% pwd
/home/dalexander/AdmixtureRuns
% ls
```

(no output—current directory is empty)

```
% ls ~/Data
hapmap3.ped  hapmap3.map
% admixture ~/Data/hapmap3.ped 3
```

(wait for it to run)

```
% ls
hapmap3.3.Q hapmap3.3.F
% ls ~/Data
hapmap3.ped  hapmap3.map
```

If you also wanted standard errors, then instead of the last command you should have used

```
% admixture -B ~/Data/hapmap3.ped 3
```

This will perform point estimation and then will also use a bootstrapping procedure to calculate the standard errors. Note that (point-estimation & bootstrapping) takes considerably longer than point-estimation alone, so you will have to be patient. Eventually it will finish, yielding point estimates and standard errors:

```
% ls
hapmap3.3.Q hapmap3.3.F hapmap3.3.Q_se
```

The "_se" file is in the same unadorned file format as the point estimates.

If your analyses are taking a long time, first consider why. Is your dataset huge? Do you really need to analyze all the markers, or can you thin the marker set (as described in Section 2.3)? If you really do need to analyze all the markers, or if your analysis is slowish for other reasons (very large K, extensive cross-validation or bootstrapping) you might consider running ADMIXTURE in multithreaded mode. If your computer has four processors, for example, you could use a command like

```
% admixture ~Data/huge_dataset.bed 3 -j4
```

to split ADMIXTURE's work among four threads—which can make ADMIXTURE run almost four times as fast.

2 Reference

2.1 How do I choose the correct value for K?

Use ADMIXTURE's cross-validation procedure. A good value of K will exhibit a low cross-validation error compared to other K values. Cross-validation is enabled by simply adding the `--cv` flag to the ADMIXTURE command line. In this default setting, the cross-validation procedure will do 10 repetitions, each time holding out 10% of the genotypes at random. The cross-validation error is reported in the screen output. For example, if in our bash shell we ran

```
% for K in 1 2 3 4 5;  \
    do admixture --cv hapmap3.bed $K | tee log${K}.out; done
```

(i.e., ran ADMIXTURE with cross-validation for K values 1,2,3,4 and 5), then we could quickly view the CV errors:

```
%  grep -h CV log*.out
CV error (K=1): 0.58135 (0.00015)
CV error (K=2): 0.53949 (0.00014)
CV error (K=3): 0.53637 (0.00012)
CV error (K=4): 0.53689 (0.00013)
CV error (K=5): 0.53740 (0.00013)
```

where the number in parentheses is the standard error of the cross-validation error estimate. We can easily plot these values for comparison, as in Figure 1, which makes it fairly clear that $K = 3$ is a sensible modeling choice.

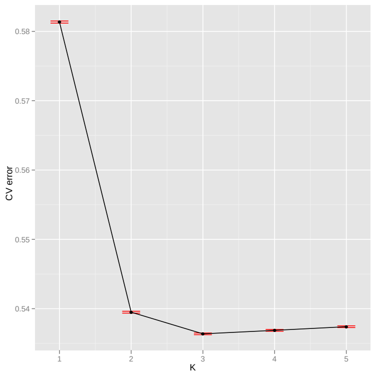

Figure 1: Cross-validation plot for the hapmap3 dataset

2.1.1 Background

Those familiar with STRUCTURE know that it provides a means of identifying the "best" value for K, the number of populations, based on computing the *model evidence* for each possible K value. The model evidence for K is defined as

$$\Pr(G \mid K) = \int \Pr(G \mid Q, F, K) \Pr(Q, F \mid K) \, dQ \, dF$$

and STRUCTURE approximates the integral via Monte Carlo. The evidence is then combined with a noninformative prior via Bayes Theorem to arrive at the posterior probabilities $\Pr(K \mid G)$.

ADMIXTURE does not attempt to estimate the model evidence. Rather, ADMIXTURE includes a *cross-validation* procedure that allows the user to identify the value of K for which the model has best predictive accuracy, as determined by "holding out" data points. Our approach is conceptually similar to that used by fastPHASE [3], and has heritage

tracing back to Wold's method [4] for cross-validating the number of components in PCA models.

More precisely, our cross-validation procedure masks (i.e. converts to "MISSING") a fraction of the genotypes in the dataset from the estimation procedure, so that a masked dataset $\tilde{G}$ is used to calculate estimates $\tilde{\theta} = (\tilde{Q}, \tilde{F})$. Each masked genotype g_{ij} is predicted by $\mathrm{E}[g_{ij}|\tilde{Q}, \tilde{F}] = 2\sum_k \tilde{q}_{ik}\tilde{f}_{kj}$ and the prediction error is calculated as the mean squared difference between the true g_{ij} and its predictor $\tilde{g}_{ij}$ over the masked data points. The procedure is repeated a number of times and the results averaged to give the cross-validation error.

2.2 How do I plot the Q estimates?

The Q estimates are output as a simple matrix, so it is easy to make figures like Figure 1 from our paper using the `read.table` and `plot` commands in R. To make the stacked bar-charts that you may have seen elsewhere, use the `barplot` command. For example, assuming we have the file `hapmap3.3.Q` to analyze, the following R commands

```
> tbl=read.table("hapmap3.3.Q")
> barplot(t(as.matrix(tbl)), col=rainbow(3),
             xlab="Individual #", ylab="Ancestry", border=NA)
```

generate the plot in Figure 2.

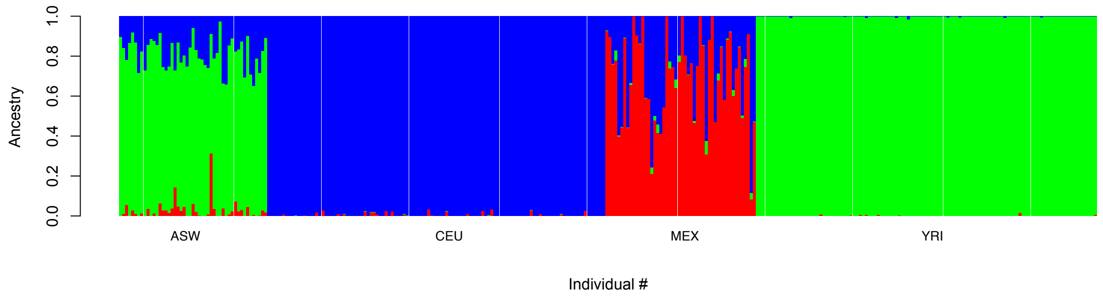

Figure 2: Stacked barplot generated in R

2.3 Do I need to thin the marker set for linkage disequilibrium?

We tend to believe this is a good idea, since our model does not explicitly take LD into consideration, and since enormous data sets take more time to analyze. It is impossible to

"remove" all LD, especially in recently-admixed populations, which have a high degree of "admixture LD". Two approaches to mitigating the effects of LD are to include markers that are separated from each other by a certain genetic distance, or to thin the markers according the observed sample correlation coefficients. The easiest way is the latter, using the `--indep-pairwise` option of PLINK. For example, if we start with a file rawData.bed, we could use the following commands to prune according to a correlation threshold and store the pruned dataset in prunedData.bed:

```
% plink --bfile rawData --indep-pairwise 50 10 0.1
```

(output indicating number of SNPs targeted for inclusion/exclusion)

```
% plink --bfile rawData --extract plink.prune.in --make-bed --out prunedData
```

Specifically, the first command targets for removal each SNP that has an R^2 value of greater than 0.1 with any other SNP within a 50-SNP sliding window (advanced by 10 SNPs each time). The second command copies the remaining (untargetted) SNPs to prunedData.bed.

This approach is imperfect but seems to work well in practice. Please read our paper for more information.

2.4 How many markers do I need to supply to ADMIXTURE?

This depends on how genetically differentiated your populations are, and on what you plan to do with the estimates. It has been noted elsewhere [2] that the number of markers needed to resolve populations in this kind of analysis is inversely proportional to the genetic distance (F_{ST}) betweeen the populations.

It is also noted in that paper that more markers are needed to perform adequate GWAS correction than are needed to simply observe the population structure.

As a rule of thumb, we have found that 10,000 markers suffice to perform GWAS correction for continentally separated populations (for example, African, Asian, and European populations $F_{ST} > .05$) while more like 100,000 markers are necessary when the populations are within a continent (Europe, for instance, $F_{ST} < 0.01$).

2.5 How do I change the random seed?'

To change the random seed to 12345, for example, you can use:

```
% admixture -s 12345 myFile.ped 3
```

or if you want the random seed to be generated from the current time, use

```
% admixture -s time myFile.ped 3
```

2.6 Optimization Methods

The default optimization method used by ADMIXTURE is a block relaxation algorithm. An alternative method, an EM algorithm (identical to that implemented by the program FRAPPE) is also available. To use this alternative algorithm, use the `-m` switch to choose the *method*:

```
% admixture -m EM ~/Data/hapmap3.ped 3
```

The convergence of the block relaxation algorithm is generally much faster, so there is no particular reason to use the EM algorithm.

2.7 Termination criteria

The default termination criterion is to stop when the log-likelihood increases by less than $\epsilon = 10^{-4}$ between iterations. A different termination criterion can be specified. A termination criterion is defined as either (1) a convergence criterion ϵ for the log-likelihood ($\epsilon < 1$) , or (2) a maximum number N of iterations, $N \geq 1$. ϵ or N is given after the flag `-C` in order to specify a desired termination criterion. For example, to stop when the log-likelihood change between iterations falls below 0.1, one could use

```
% admixture -C 0.1 ~/Data/hapmap3.ped 3
```

Or, to stop the algorithm after 10 iterations one could use

```
% admixture -C 10 ~/Data/hapmap3.ped 3
```

Note that this "overloaded" use of `-C` flag makes it impossible to use an ϵ greater than one. This doesn't seem a major limitation to us at present, since in general a small value is desired for ϵ.

The `-C` flag designates the *major termination criterion*, i.e. the criterion for stopping the point estimation algorithm. In our paper we point out that we use a looser convergence criterion for re-estimation within bootstrap resamples. This *minor termination criterion* is specified in the same manner, but using the `-c` flag (lowercase). The default value for this is 3 (i.e., 3 iterations).

2.8 Acceleration methods

By default, ADMIXTURE uses the quasi-Newton convergence acceleration method described in the paper, with $q = 3$ secant conditions. To use quasi-Newton acceleration with a different number of secant conditions—for example, 2—use the `-a` flag as follows:

```
% admixture -a qn2  ~/Data/hapmap3.ped 3
```

To turn off acceleration entirely, use `-a none`.

2.9 Bootstrapping

ADMIXTURE estimates parameter standard errors using bootstrapping. As described in "Quick Start", the basic way to get bootstrap standard errors is to include the `-B` flag when you run ADMIXTURE:

```
% admixture -B myInput.geno 3
```

This uses the default of 200 bootstrap replicates. Perhaps you want more? Try

```
% admixture -B2000 myInput.geno 3
```

to use 2000 bootstrap replicates. Naturally, this will take longer to run. Don't forget that you need to have a PLINK `.map` file in place in order to do bootstrapping.

2.10 Supervised analysis

Estimating F and Q from the SNP matrix G, without any additional information, can be viewed as an *unsupervised* learning problem. However it is not uncommon that some or all of the individuals in our data sample will have known ancestries, allowing us to set some rows in the matrix Q to known constants. This allows more accurate estimation of the ancestries of the remaining individuals, and of the ancestral allele frequencies. Viewing these *reference* individuals as training samples, the problem is transformed into a *supervised* learning problem.

Supervised learning mode is enabled with the flag `--supervised` and requires an additional file with a `.pop` suffix, specifying the ancestries of the reference individuals. It is assumed that all reference samples have 100% ancestry from some ancestral population. Each line of the `.pop` file is a string designating a population or is empty (indicating the individual is of unknown ancestry, to be estimated).

2.11 Multithreaded mode

To split ADMIXTURE's work among N threads, you may append the flag `-jN` to your ADMIXTURE command. The core algorithms will run up to N times as fast, presuming you have at least N processors.

2.12 Penalized estimation

We have recently been exploring penalized maximum likelihood estimation as an alternative to pure maximum likelihood. Maximum likelihood should be suitable for almost all users, but those with very small datasets or very large K values can experiment with penalized estimation, which maximizes a modified objective function, in practice introducing sparsity in the Q parameter estimates, eliminating those parameters with low explanatory power. The penalty function we implement is

$$\mathcal{P}(Q) = \sum_{i,k} \frac{\log(1 + q_{ik}/\epsilon)}{\log(1 + 1/\epsilon)},$$

and the modified objective function is $\mathcal{G}(Q, F) = \mathcal{L}(Q, F) - \lambda\mathcal{P}(Q)$, where $\mathcal{L}(Q, F)$ denotes the log-likelihood function.. Lambda and epsilon are chosen by the user with the `-l` and `-e` flags. For example, penalized estimation can be invoked using

```
% admixture myInput.geno 3 -l 500 -e 0.1
```

Larger lambda values and larger epsilon values (which must remain less than one) introduce more sparsity in Q estimates.

3 Citation and further information

If you use ADMIXTURE, please cite our paper [1].

Bibtex code:

```
@article{alexander2009admixture,
  title={{Fast model-based estimation of ancestry in unrelated individuals}},
  author={Alexander, D.H. and Novembre, J. and Lange, K.},
  journal={Genome Research},
  volume={19},
  pages={1655--1664},
  year={2009},
```

```
publisher={Cold Spring Harbor Lab},
doi={10.1101/gr.094052.109}}
```

Contact Dave at `dalexander@ucla.edu` with further questions.

References

[1] D.H. Alexander, J. Novembre, and K. Lange. Fast model-based estimation of ancestry in unrelated individuals. *Genome Research*, 19:1655–1664, 2009.

[2] N. Patterson, AL Price, and D. Reich. Population structure and eigenanalysis. *PLoS Genet*, 2(12):e190, 2006.

[3] P. Scheet and M. Stephens. A fast and flexible statistical model for large-scale population genotype data: applications to inferring missing genotypes and haplotypic phase. *The American Journal of Human Genetics*, 78(4):629–644, 2006.

[4] S. Wold. Cross-validatory estimation of the number of components in factor and principal components models. *Technometrics*, 20(4):397–405, 1978.
